# Immunotherapy Treatment for Triple Negative Breast Cancer

**DOI:** 10.3390/ph14080763

**Published:** 2021-08-04

**Authors:** Elizabeth R. Berger, Tristen Park, Angeleke Saridakis, Mehra Golshan, Rachel A. Greenup, Nita Ahuja

**Affiliations:** Department of Surgery, Yale University School of Medicine, 310 Cedar Street, 118 Lauder Hall, New Haven, CT 06511, USA; tristen.park@yale.edu (T.P.); Angeleke.Saridakis@yale.edu (A.S.); Mehra.Golshan@yale.edu (M.G.); Rachel.Greenup@yale.edu (R.A.G.); Nita.Ahuja@yale.edu (N.A.)

**Keywords:** triple-negative breast cancer, immunotherapy, treatment algorithms

## Abstract

Triple-negative breast cancer (TNBC) is considered one of the highest-risk subtypes of breast cancer and has dismal prognosis. Local recurrence rate after standard therapy in the early breast cancer setting can be upwards to 72% in 5 years, and in the metastatic setting, the 5-year overall survival is 12%. Due to the lack of receptor expression, there has been a paucity of targeted therapeutics available, with chemotherapy being the primary option for systemic treatment in both the neoadjuvant and metastatic setting. More recently, immunotherapy has revolutionized the landscape of cancer treatment, particularly immune checkpoint inhibitor (ICI) therapy, with FDA approval in over 20 types of cancer since 2011. Compared to other cancer types, breast cancer has been traditionally thought of as being immunologically cold; however, TNBC has demonstrated the most promise with immunotherapy use, a timely discovery due to its lack of targeted therapy options. In this review, we summarize the trials using checkpoint therapy in early and metastatic TNBC, as well as the development of biomarkers and the importance of immune related adverse events (IRAEs), in this disease process.

## 1. Introduction

With the number of predicted breast-cancer cases in the United States reaching 250,000 in the year 2020, it is estimated that 10–15% of these newly diagnosed cases will be triple-negative breast cancer (TNBC) [1,2,3,4]. TNBC lacks the expression of the estrogen receptor (ER) and progesterone receptor (PR) and human epidermal growth factor receptor 2 (Her2) [5,6]. It is associated with earlier age of onset, higher rates of recurrence, increased risk of visceral metastasis, and poorer prognosis when compared to hormone sensitive subtypes [7,8,9,10,11]. Additionally, TNBC disproportionately affects young women (<40 years) and has a higher incidence among African American and Hispanic women when compared with other breast-cancer subtypes [12,13,14,15]. The lower survival rate is attributable to various aspects of the disease, such as higher genomic instability, higher-grade and mitotic index, mutations of the p53 and BRCA1 gene, and lymphatic dissemination [16,17]. TNBC also tend to be more aggressive and larger at time of diagnosis, due to more difficulty with detection by conventional imaging [7,18,19,20]. Some of the early gene-expression profiling studies identified and categorized breast cancers into luminal-like, Her-2-positive, basal-like, and normal breast-like [5]. Luminal A subtype is hormone receptor-positive and is known to have the best prognosis, followed by the Luminal B subtype. In contrast, “basal-like” breast cancers often correlate histologically and clinically with TNBC and tend to have poor relapse-free survival rates and the worst prognosis [21]. Among TNBC, the lack of expression of measured receptors, defined molecular targets, and significant heterogeneity make chemotherapy the mainstay of systemic treatment to date; targeted therapeutic agents specific to TNBC remain under investigation and, until recently, have not demonstrated significantly improved survival in patients [22,23,24].Thus, historically, there have been limited treatment options for this high-risk breast-cancer phenotype [18,25].

Immunotherapy has prolonged survival in other solid tumors, including melanoma, lung, and kidney cancers with the most successful immunotherapeutic agents consisting of immune checkpoint inhibitors (ICIs). ICIs block immune checkpoints or immunosuppressive receptors, such as cytotoxic-T-lymphocyte-associated protein 4 (CTLA-4) and programmed death receptor-1 (PD-1) to improve the cytotoxicity and proliferative capacity of tumor-infiltrating lymphocytes (TILs) [26,27,28,29,30,31,32,33,34,35]. T-cells have the CTLA-4 receptor, which acts as an immune checkpoint, helping to downregulate the immune system. Alternatively, PD-1, which is expressed on the surface of T-cells, suppresses the autoimmunity of T-cells and is a marker for T-cell exhaustion [36,37]. When a person develops a malignancy, tumor antigens create an upregulation by T-cells of multiple inhibitory receptors, which can lead to impaired tumor recognition [38]. Solid organ tumor cells express programmed death-ligand 1 (PD-L1) that attaches to the PD-1 receptors on T-cells, resulting in challenges of the immune system to recognize proliferating tumor cells [39]. Immunotherapies, such a pembrolizumab, Nivolumab, and Atezolizumab, help to augment antitumor immunity by either targeting PD-1 receptors on T-cells or PD-L1 ligand on tumor cells [40].

Unlike other solid organ cancers, breast cancer has long been considered more immunologically “cold” with low T-cell infiltration. However, several lines of evidence have demonstrated the immune system’s role in the prognosis and outcomes of TNBC. First, TNBC has more TILs, with several studies demonstrating that these are associated with a higher response to ICIs in other tumors and improved prognosis in early stage TNBC [26,27,41,42,43,44,45]. Second, studies suggest that there is significant activation of inhibitory immune checkpoints with higher PD-L1 expression in TNBC compared to luminal subtypes. As discussed, the binding of PD-1 to its ligand (PD-L1) activates the PD-1/PD-L1 axis, suppressing the immune response [46]. PD-L1 expression inhibits immune cells in the innate and adaptive immune system, including T-cells, B-cells, natural killer cells, dendritic cells, and macrophages [47]. This high level of PD-L1 expression in TNBC provides direct targets for ICIs, including anti-PD-1 therapies [28,46,48]. Finally, TNBC possesses a higher rate of nonsynonymous somatic mutations compared to other subtypes; this increase in tumor mutational burden (TNB) generates more tumor-specific neoantigens which can be a putative target for the immune system [49].

In this review, we focus on the role of immune checkpoint inhibitors in early stage and metastatic triple-negative breast cancer either in the neoadjuvant or adjuvant settings. In addition, we summarize the effects of combination treatments with chemotherapy and immune checkpoint inhibitors, as well as recent and ongoing trials attempting to elucidate the role of promising therapeutic strategies that target specific subsets of TNBC. We briefly outline other potentially immunotherapy strategies and future directions.

## 2. Heterogeneity of Triple-Negative Breast Cancer

Triple-negative breast cancer is uniquely heterogeneous at the clinical, histologic, and molecular level. Breast tumors that do not overexpress the estrogen, progesterone, or Her2neu receptor are histologically known as TNBC. The American College of Pathology, the American Society of Clinical Oncology, and the St. Gallen have established that ER and PR negativity are defined by 1% positivity of either receptor [50,51]. Molecularly, TNBC has been classified into six subtypes, namely basal-like subtypes (BL1 and BL2), mesenchymal (M), mesenchymal stem-like (MSL), immunomodulatory (IM), and luminal androgen receptor (LAR) [52]. A study by Lehmann and colleagues analyzed 587 TNBCs by gene-expression profiling and identified these specific six subtypes which are now used in subdividing this particular breast cancer [52]. This distinct heterogeneity contributes to limited therapeutic treatment options for TNBC.

## 3. Monoclonal Antibodies Targeting Immune Checkpoints in Metastatic Setting

Immune checkpoints are molecules that protect against normal tissue damage caused by over-activity of T-cells [9]. PD-1 and its ligand PD-L1, the most widely studied immune checkpoint receptors in the treatment of breast cancer, are expressed on activated T-cells, B-lymphocytes, and natural killer cells and are associated with tumor immune resistance [53]. An abundancy of genes involved in immune cell processes and high levels of tumor-infiltrating lymphocytes implying high immunogenicity for the IM subtype of TNBC [49]. These characteristics suggest that immune checkpoint inhibitors (ICIs) are feasible therapeutic agents for TNBC. ICIs were first investigated amongst metastatic TNBC patients as a monotherapy. Subsequently, there have been trials to investigate ICIs in combination with chemotherapy agents to enhance response rates, as well as using ICIs in the neoadjuvant setting.

### 3.1. PD-1 Inhibitors

#### Pembrolizumab

One of the most studied ICIs, pembrolizumab is a humanized IgG4 antibody targeting PD-1. Pembrolizumab first gained initial FDA approval for unresectable or metastatic melanoma in 2014 [37] and has more recently shown promise in other solid organ cancers [54]. In breast cancer, pembrolizumab was first tested as a monotherapy in the initial phase Ib KEYNOTE-012 study of 32 patients with both pretreated with chemotherapy and treatment-naïve PD-L1-positive TNBC [55]. It demonstrated an encouraging overall response rate (ORR) of 18.5%, which lead to the first ICI approval in TNBC. The following large phase II KEYNOTE-086 study did not demonstrate as impressive ORR-in 170 patients with a PD-L1-unselected pretreated tumor the ORR was only 5.3 In the same study, 84 patients who were treatment-naïve on the trial, the ORR was 21.4% suggesting that ICIs have greater efficacy in the first-line metastatic setting [56]. A phase III study, the KEYNOTE-119 trial, demonstrated similar findings as KEYNOTE-086 with pretreated metastatic TNBC not showing any improvement in progression-free (PFS) or overall survival (OS) with single-agent pembrolizumab versus single-agent chemotherapy [57]. Monotherapy in early stage TNBC has not been evaluated, due to tempered response of monotherapy in the metastatic setting.

### 3.2. PD-L1 Inhibitors

#### Atezolizumab/Avelumab

A humanized IgG1 antibody targeting PD-L1, called Atezolizumab, has also been explored as ICI monotherapy in metastatic TNBC. It selectively targets PD-L1 to prevent interaction with the receptors PD-1 and B7-1, reversing T-cell suppression [58]. In a phase 1 trial, Atezolizumab led to an ORR of 10% in 115 pretreated patients, with no responses seen in the PD-L1 negative subgroup [59]. The phase 1b JAVELIN trial examined Atezolizumab as a monotherapy and demonstrated a ORR of only 5.2% in 58 heavily pretreated patients [60]. With particularly low response rates in the pretreated metastatic disease groups, these trials demonstrate the limited efficacy as a single agent in metastatic TNBC.

## 4. Chemotherapeutic Agents Used in Combination with Immunotherapy

It is generally accepted that most TNBC is chemotherapy-sensitive, but the optimal treatment regimen continues to be investigated. Most chemotherapy regimens include anthracyclines, taxanes, and/or platinum compounds, dose-dense AC (doxorubicin/cyclophosphamide), or TC (docetaxel/cyclophosphamide). The addition of platinum to standard chemotherapy has shown to increase the pathologic complete response rate [61,62]. Multiple guidelines support the use of chemotherapy in the neoadjuvant setting for early stage TNBC [63]. Often a surrogate endpoint for clinical trials, pathological complete response after neoadjuvant chemotherapy is predictive of long-term survival outcomes [64]. Administration of anthracycline and taxane-based chemotherapy sequentially is the most common neoadjuvant approach, with the consideration of adding carboplatin, as it has been demonstrated to improve the pathologic complete response (pCR) rate [65].

With the introduction of ICIs, the therapeutic landscape has changed. Generally, ICI monotherapy was effective against metastatic TNBC when there was limited disease present; however, in women with advanced disease and a high metastatic tumor burden, there was minimal or no response. Therefore, most studies began to focus on combination therapy of ICI with chemotherapy. The hypothesis was that chemotherapy could augment tumor-antigen release and antitumor responses to ICI. Specifically, taxanes were thought to have the potential to activate toll-like receptor activity and promote dendritic-cell activity [66]. The KEYNOTE-355 trial reported first-line chemotherapy with pembrolizumab significantly improved PFS compared with chemotherapy in patients with metastatic TNBC expressing PD-L1 [67].

The Impassion130 trial demonstrated significantly prolonged median PFS with a regimen of atezolizumab plus nab-paclitaxel compared to placebo plus nab-paclitaxel. (7.2 months and 5.5 months respectively); the PD-L1 positive immune cells subgroup demonstrated an even more dramatic difference in PFS. The difference between PFS of atezolizumab and control groups was 7.5 months vs. 5.0 months) [58]. There was no difference in OS between these two groups when not selected for PD-L1 positivity. When the PD-L1 positive group was analyzed, OS was improved by 7 months in the atezolizumab group (25.0 vs. 18.0 months). With these promising results, the Food and Drug Administration granted accelerated approval to atezolizumab in March of 2019. Thus, the combination therapy of immunotherapy and chemotherapy became standard of care in patients with unresectable locally advanced or metastatic PD-L1 positive TNBC [26]. There is an ongoing Impassion131 trial which is evaluating atezolizumab in combination with paclitaxel compared with placebo and paclitaxel for patients with previously untreated inoperable locally advanced or metastatic TNBC. Early results show that atezolizumab with paclitaxel failed to improve outcomes for patients [68].

## 5. Early Stage Chemotherapy Combination Regimens

Due to the promising results in the metastatic setting of combination chemotherapy and ICIs, studies have now been conducted in early stages of TNBC. They have demonstrated preliminary success thus far. In the I-SPY 2 trial, patients with stage II/III disease treated with combination chemotherapy and pembrolizumab had estimated the pCR rate to be nearly three times that of those individuals with chemotherapy alone [55]. The KEYNOTE-522 trial also demonstrated improved pCR rates (51.2% to 64.8%) and 18-month event-free survival (EFS) (85.3% to 91.3%) when pembrolizumab was delivered in combination with chemotherapy in both the neoadjuvant and adjuvant settings [27,69]. In contrast to the aforementioned findings, the NeoTRIPaPDl1 study demonstrated that standard chemotherapy in combination with atezolizumab did not significantly impact pCR rates in patients with early stage high-risk or locally advanced TNBC [70]. High-risk disease was defined as disease with high proliferation or grade. The contrasting results of these two studies may be explained by chemotherapy backbones or due to differences in the ICI activity, given that PD-1 inhibitors but not PD-LA inhibitors block PD-L2 inhibitory signaling [27,71,72].

There are several ongoing early stage disease trials that will further clarify the efficacy of ICIs in the neoadjuvant setting for TNBC and whether these agents should be utilized as adjuvant treatments. Two key trials—namely SWOG S1418 and the A-brave trial—are investigating if adjuvant anti-PD-1/L1 therapy prolongs event-free survival (EFS) or disease-free survival (DFS). Concurrently, two large trials are investigating if the addition of atezolizumab to both neoadjuvant and adjuvant therapy prolongs EFS or invasive DFS-the NSABP B-59 trial and the Impassion030 trial.

## 6. Phase 3 Randomized Controlled Trials

### 6.1. KEYNOTE-119

KEYNOTE-119 is a phase 3 trial that compared pembrolizumab with chemotherapy for second-line or third-line treatment of patients with metastatic TNBC. The trial included 1098 patients who were randomly assigned to receive either pembrolizumab or chemotherapy. The primary endpoints were OS in PD-L1 positive patients and all patients. In the PD-L1-positive patients, the median OS was 12.7 months in the pembrolizumab group and 11.6 months for the chemotherapy group (0.057). In the overall population, the median OS was 9.9 months for the pembrolizumab group and 10.8 months for the chemotherapy group (non-significant). The most common adverse events were anemia and neutropenia [57].

### 6.2. KEYNOTE-355

Based upon the findings of the KEYNOTE-119 trial, this trial is a phase 3 trial that compared pembrolizumab with chemotherapy versus placebo with chemotherapy untreated locally recurrent inoperable or metastatic TNBC. The trial included 1372 patients who were randomly assigned to the two treatment groups and the primary endpoint was PFS. IN the PD-L1 positive patients, the PFS in the pembrolizumab group was 9.7 months and 5.6 months in the placebo group (*p* = 0.0012). Among all patients, the median PFS was 7.5 months compared to 5.6 months. The pembrolizumab treatment effect increased with PD-L1 enrichment. Adverse events were seen in 68% of the pembrolizumab group and 67% in the placebo group [71].

### 6.3. KEYNOTE-522

Previous trials demonstrated promising results with pembrolizumab in addition to chemotherapy. As a result, the KEYNOTE-522 study was a phase 3 trial that compared pembrolizumab with chemotherapy versus placebo with chemotherapy in the neoadjuvant setting among untreated stage II or stage II TNBC. The two groups received additional adjuvant cycles of either pembrolizumab or placebo and both groups received adjuvant chemotherapy. The trial included 602 patients who were randomized into the two treatment groups and the primary pCR at the time of definitive surgery and event-free survival. The pCR was 64.8% in the pembrolizumab group versus 51.2% in the placebo group and there were 7.4% of patients in the pembrolizumab group who had disease progression prior to surgery vs. 11.8% in the placebo group. The adverse events were similar across the two groups; 78.0% versus 73.0%, respectively. Unlike trials in the metastatic setting, there was an improvement in the pCR rate compared to chemotherapy alone, irrespective of PD-L1 levels; however, the PD-L1+ group had the highest absolute pCR of 81.7%.

### 6.4. KEYNOTE-242

KEYNOTE-242 is a phase 3 trial that is comparing pembrolizumab for 1 year in the adjuvant setting versus placebo in patients with ≥1 cm of residual invasive cancer and/or positive lymph nodes after neoadjuvant chemotherapy. The trial includes TNBC or low estrogen-receptor (ER0-positive and/or Her2 borderline breast cancers). The primary outcomes are DFS and OS. The trial is ongoing and continues to accrue patients (NCT02954874).

### 6.5. Impassion130

Impassion130 is a phase 3 trial that compared atezolizumab in combination with nab-paclitaxel or a placebo in patients with untreated metastatic TNBC. The trial included 451 patients in each group and was randomized. The intervention was continued until disease progression or an unacceptable level of toxic effects occurred. The primary endpoints were PFS and OS. The median PFS was 7.2 months with atezolizumab, as compared with 5.5 months (*p* = 0.01) with placebo with the median OS being 21.3 months compared with 17.6 months (*p* = 0.01), respectively within the two groups. Among the patients with PD-L1 positive tumors, the median OS was 25.0 months and 15.5 months, respectively. Adverse events that led to the discontinuation of any agent occurred in 15.9% of the patients who received atezolizumab and 8.2% of those who received a placebo [69].

### 6.6. Impassion030

Another ongoing phase 3 trial, the Impassion030, compares atezolizumab in combination with standard arthracycline/taxane adjuvant chemotherapy in early TNBC patients. There are 2300 patients included who have operable stage II or III TNBC. They were randomized and stratified based upon type of surgery, nodal status, and PD-L1 status. The adjuvant treatment was either weekly paclitaxel for 12 weeks, followed by four doses of dose dense anthracycline and cyclophosphamide, or the same chemotherapy regimen given concomitantly with atezolizumab every 2 weeks, followed by every third week of maintenance atezolizumab until completion of 1 year of therapy. DFS is the primary end point [72]. Active recruitment to this trial continues, and no results have been released (NCT03498716).

### 6.7. Impassion031

Impassion031 is a phase 3 trial, similar to Impassion030, that compared atezolizumab versus placebo combined with nab-paclitaxel followed by doxorubicin plus cyclophosphamide as neoadjuvant treatment for early stage TNBC. The trial included 333 patients who were randomly assigned to either atezolizumab plus chemotherapy or placebo plus chemotherapy, and the primary endpoints were pCR in all patients and in PD-L1 positive patients. In all patients, the pCR with atezolizumab was 58% vs. 41% in the placebo group (*p* = 0.004). In the PD-L1 positive patients, the difference was more pronounced—with 69% achieving a pCR in the atezolizumab group and 49% in the placebo group. The adverse events were balanced across the two groups [73].

### 6.8. ISPY-2

The I-SPY2 study is an ongoing open-label, multicenter, adaptively randomized phase 2 platform trial for high-risk, stage II/III breast cancer patients that evaluates multiple investigational arms in parallel. In this hypothetical confirmatory phase 3 trial, patients are randomized to receive taxane- and anthracycline-based neoadjuvant chemotherapy with or without pembrolizumab, followed by definitive surgery. There were 250 people included in the final analysis, and the primary endpoint was pathologic complete response. In the TNBC cohort, the pCR rates were 44% versus 17% for the pembrolizumab vs. control, respectively. Adverse events included immune-related endocrinopathies, notably thyroid abnormalities, and adrenal insufficiency [55].

### 6.9. NSABP B–59/GBG 96-GeparDouze

NSABP B-59 is a phase 3 trial that is comparing neoadjuvant chemotherapy with atezolizumab or placebo in patients with early stage TNBC followed by adjuvant atezolizumab or placebo. The primary endpoints are EFS and pCR in the breast and lymph nodes. Patients are actively being recruited to this trial currently [74] (NCT03281954).

## 7. Other Novel Immunotherapy Strategies

Already discussed, CTLA-4 acts earlier in the T-cell activation process and is significant contributes to the suppressive mechanism of the regulator T-cell (Treg) [75]. Recent research in both lung cancer and melanoma has revealed that the exhaustion of T-regulatory cells by anti-CTLA-4 therapy is one of the main reasons leading to therapeutic responses [76]. The combination of PD-1 abd anti-CTLA- antibodies, Nivolumab and ipilimumab, respectively, have demonstrated better responses in melanoma and lung cancer compared to nivolumab or chemotherapy alone [77,78]. There are ongoing phase I/II trials exploring the combinatorial effect of CTLA-/PD-1 antibodies in metastatic TNBC (NCT02536794).

BRCA-1-mutated tumors have been demonstrated to be deficient in DNA-repair, as well as 25% of sporadic breast cancers. PARP (poly (ADP-ribose) polymerase) is a nuclear enzyme that helps repair DNA single-strand breaks and is highly expressed in more than 90% of TNBC [79]. Polymerase inhibitors (PARPi) that target these recombination repair pathways and have been found to be effective in the treatment of BRCA1/2 mutation carriers with TNBC are being used in combination with immune checkpoint blockade to trigger a stronger antitumor immune response. PARPi-induced cell death causes the release of tumor antigens that activate infiltrating T-cells. PARPi also upregulate PD-L1 expression in animal models, further strengthening the rationale for combining treatment with PD1/PD-L1 inhibitors [80,81]. (NCT03281954). There are ongoing trials that are currently in the recruitment phase to investigate the use of PARPi in treating TNBC.

Another novel strategy in cancer immunotherapy are cancer vaccines, with the potential to illicit an immune response against tumor-specific and tumor-associated antigens. Ongoing trials in TNBC are investigating using tumor vaccines or oncolytic viruses with csfg312blockade against TNBC [75] and poly (ADP-Ribose).

## 8. Immunotherapy Challenges

There are many promising results in past and ongoing trials using immunotherapy for treatment in TNBC. However, there are ongoing challenges with these treatments, including development of biomarkers for optimal patient selection and immune-related adverse events (IRAEs) [82,83].

### Biomarker Development

**Figure 1:**
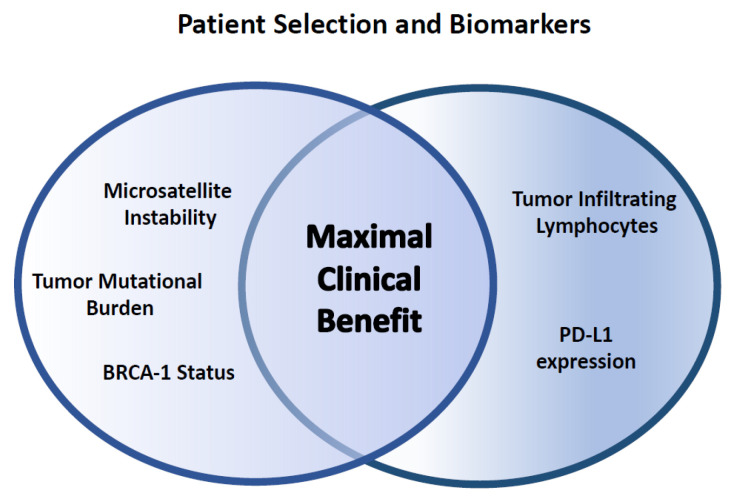
Biomarker development for optimization of immunotherapy use in patients.

Enriching the population with the development of biomarkers to determine who may derive benefit from ICI is an area of active investigation (Figure 1). One of the most established biomarkers includes expression of PD-L1: TNBC patients with PD-L1 expression on immune cells received most clinical benefit in the Impassion130 trial. In the KEYNOTE 522 trial, incremental improvement of pCR (~15%) with addition of ICI was not dependent on PD-L1 expression; however, PD-L1+ population had the highest overall pCR rate at 81.7%. PD-L1 expression on macrophages and tumor cells has been demonstrated to be a possible predictor for pCR in other neoadjuvant ICI studies [84].

Quantitative and qualitative TIL levels have also shown promise in predicting patient response to ICI. In the Impassion130 trial, metastatic TNBC patients with higher CD8+ TILs demonstrated increased PFS and OS when treated with chemo-immunotherapy. In early stage TNBC patients treated with pembrolizumab-based combination therapy, higher quantities of TILs and PD-L1 expression was associated with a higher pCR rates and improved ORR [43]. Further refining the TIL population is also being actively studied; a TIL signature of CD8+ tissue-resident memory T (T_RM_) cell differentiation expressing high levels of immune checkpoint molecules has been associated with survival in an early TNBC population and was better prognosticator than the CD8+ TIL population alone [85].

Other biomarkers that are under investigation include tumor mutation burden (TMB), microsatellite instability (MSI), and BRCA status. TNBC harbors more TMB or non-synonymous somatic mutations than other breast-cancer subtypes. In other cancers, a higher TMB is correlated with improved response to ICI therapy. One study has shown that higher levels of TMB with high TIL infiltrates have been shown to be correlated with better prognosis in TNBC [86]. In addition, adoptive transfer of TILs specific for these somatic mutations have mediated durable complete responses in the metastatic setting in breast and other epithelial cancers in clinical trials [87,88]. Tumors with high levels of MSI have been shown to be highly responsive to ICI-based immunotherapy, and pembrolizumab has been uniquely FDA approved for any metastatic solid tumor that harbors high-MSI. The incidence of MSI in breast cancer still needs to be fully evaluated, though the existing studies have shown a low incidence of <2% thus far [89]. Interestingly, BRCA1 mutations are predisposed to TNBC, and BRCA1-deficient TNBCs are also known to have higher levels of TILs, higher levels of somatic mutations, and higher levels of immunomodulatory genes which can represent a subgroup that would benefit from immunotherapeutic approaches [90]. Immunotherapy has also been explored and used in BRCA-associated ovarian cancers. With the advent of poly (ADP-ribose) polymerase (PARP) inhibitors, outcomes have significantly improved for BRCA-associated ovarian cancers [91]. There are many ongoing trials investigating the use of other immunotherapy agents for ovarian cancers.

## 9. Immune-Related Adverse Events (IRAEs)

Immunotherapy hyper-activates the immune system, causing a wide variety of toxicities called immune-related adverse events (IRAEs) [82,83]. IRAEs can vary from mild flu-like symptoms to more serious manifestations, such as pneumonitis, colitis, hepatitis, and endocrinopathies such as type 1 diabetes and adrenal insufficiency, which may result in the need for lifelong replacement therapies. The incidence of most of these serious manifestations are less than 5%; however, when they do occur, it requires immediate and significant attention. There is a wide variability of severity and time of onset of IRAEs: some can manifest as a severe event after just one dose, while others develop months after. In addition, there appears to be higher risk of IRAEs when ICIs are combined with chemotherapy vs. monotherapy alone overall in all disease processes [92,93,94,95,96]. As the use of ICIs appears to be more efficacious in early stage breast cancer and will mainly be used in the combinatorial setting with chemotherapy, the sequalae of developing IRAEs that are permanent or severe may have significant ramifications compared to other malignancies where ICI will be used mainly as monotherapy and in the metastatic setting.

Predicting who will develop IRAEs is an active area of investigation and may include assessment of personal and/or genetic risk factors. Studies have shown that obesity, kidney disease, and personal or family history of autoimmune disease have been linked with increased risk, while other factors, such as history of steroid use or female sex, may be protective. Currently, the treatment of IRAEs include cessation of the ICI, and use of glucocorticoids and/or monoclonal ab, including TNF (infliximab) or IL-6 (Tocilizumab). Preliminary investigation on how the use of these agents affects the antitumor response of the ICI is reassuring in other malignancies, but requires longer-term follow-up [97,98,99,100].

Another challenge of immunotherapy is the wide variety of different response patterns seen across patients with what appears to be the same disease. In other solid organs such as non-small-cell lung cancer, there is a phenomenon known as “pseudo-progression”, where patients appear to be progressing, with tumor enlargement secondary to lymphocyte infiltration, prior to demonstrating regression. With ongoing investigations into immunotherapy in the TNBC setting, hopefully we will gain some answers to these ongoing questions.

## 10. Conclusions

The understanding of immune-checkpoint-based treatment in TNBC has radically changed the therapeutic options for women who have been diagnosed with this disease. Existing trial data have clearly demonstrated that ICIs should be combined with other agents to improve their benefit. It is also recognized that immunotherapy should be implemented in the first-line setting of metastatic treatment to improve overall survival rates [101]. Additional promise exists in the early stage setting; improvements in pCR rates have been shown across multiple trials, especially in the neoadjuvant setting and irrespective of PD-L1 levels. Due to this, investigation into the prediction and treatment of severe or permanent IRAEs is of particular importance among women with curative disease. Ongoing research efforts aim to identify biomarkers that better predict excellent pathologic and clinical responses, allowing for refined patient selection that justifies the side-effect risk profile. Immunotherapy has revolutionized the treatment of cancers overall in the last decade; since its first FDA approval for melanoma in 2011, there are now over 20 cancers that have an FDA approval for checkpoint therapy. With the promising evidence thus far, we anticipate checkpoint therapy approaches for TNBC to lead to a paradigm shift regarding treatment options for a very aggressive form of breast cancer.

## Data Availability

Data sharing not applicable.

## References

[1] American Cancer Society: Triple-Negative Breast Cancer. https://www.cancer.org/cancer/breast-cancer/understanding-a-breast-cancer-diagnosis/types-of-breast-cancer/triple-negative.html.

[2] Siegel R.L., Miller K.D., Jemal A. (2016). Cancer statistics, 2016. CA Cancer J. Clin..

[3] Carey L.A., Perou C.M., Livasy C.A., Dressler L.G., Cowan D., Conway K., Karaca G., Troester M.A., Tse C.K., Edmiston S. (2006). Race, breast cancer subtypes, and survival in the Carolina Breast Cancer Study. JAMA.

[4] Yang X.R., Sherman M.E., Rimm D.L., Lissowska J., Brinton L.A., Peplonska B., Hewitt S.M., Anderson W.F., Szeszenia-Dabrowska N., Bardin-Mikolajczak A. (2007). Differences in risk factors for breast cancer molecular subtypes in a population-based study. Cancer Epidemiol. Biomark. Prev..

[5] Perou C.M., Sorlie T., Eisen M.B., van de Rijn M., Jeffrey S.S., Rees C.A., Pollack J.R., Ross D.T., Johnsen H., Akslen L.A. (2000). Molecular portraits of human breast tumours. Nature.

[6] Brenton J.D., Carey L.A., Ahmed A.A., Caldas C. (2005). Molecular classification and molecular forecasting of breast cancer: Ready for clinical application?. J. Clin. Oncol..

[7] Dent R., Trudeau M., Pritchard K.I., Hanna W.M., Kahn H.K., Sawka C.A., Lickley L.A., Rawlinson E., Sun P., Narod S.A. (2007). Triple-negative breast cancer: Clinical features and patterns of recurrence. Clin. Cancer Res..

[8] Ademuyiwa F.O., Groman A., O’Connor T., Ambrosone C., Watroba N., Edge S.B. (2011). Impact of body mass index on clinical outcomes in triple-negative breast cancer. Cancer.

[9] Shi J., Liu F., Song Y. (2020). Progress: Targeted Therapy, Immunotherapy, and New Chemotherapy Strategies in Advanced Triple-Negative Breast Cancer. Cancer Manag. Res..

[10] Carey L.A., Dees E.C., Sawyer L., Gatti L., Moore D.T., Collichio F., Ollila D.W., Sartor C.I., Graham M.L., Perou C.M. (2007). The triple negative paradox: Primary tumor chemosensitivity of breast cancer subtypes. Clin. Cancer Res..

[11] Vaz-Luis I., Ottesen R.A., Hughes M.E., Mamet R., Burstein H.J., Edge S.B., Gonzalez-Angulo A.M., Moy B., Rugo H.S., Theriault R.L. (2014). Outcomes by tumor subtype and treatment pattern in women with small, node-negative breast cancer: A multi-institutional study. J. Clin. Oncol..

[12] Bauer K.R., Brown M., Cress R.D., Parise C.A., Caggiano V. (2007). Descriptive analysis of estrogen receptor (ER)-negative, progesterone receptor (PR)-negative, and HER2-negative invasive breast cancer, the so-called triple-negative phenotype: A population-based study from the California cancer Registry. Cancer.

[13] Lund M.J., Trivers K.F., Porter P.L., Coates R.J., Leyland-Jones B., Brawley O.W., Flagg E.W., O’Regan R.M., Gabram S.G., Eley J.W. (2009). Race and triple negative threats to breast cancer survival: A population-based study in Atlanta, GA. Breast Cancer Res. Treat..

[14] Stead L.A., Lash T.L., Sobieraj J.E., Chi D.D., Westrup J.L., Charlot M., Blanchard R.A., Lee J.C., King T.C., Rosenberg C.L. (2009). Triple-negative breast cancers are increased in black women regardless of age or body mass index. Breast Cancer Res..

[15] Morris G.J., Naidu S., Topham A.K., Guiles F., Xu Y., McCue P., Schwartz G.F., Park P.K., Rosenberg A.L., Brill K. (2007). Differences in breast carcinoma characteristics in newly diagnosed African-American and Caucasian patients: A single-institution compilation compared with the National Cancer Institute’s Surveillance, Epidemiology, and End Results database. Cancer.

[16] de Ruijter T.C., Veeck J., de Hoon J.P., van Engeland M., Tjan-Heijnen V.C. (2011). Characteristics of triple-negative breast cancer. J. Cancer Res. Clin. Oncol..

[17] Irvin W.J., Carey L.A. (2008). What is triple-negative breast cancer?. Eur. J. Cancer.

[18] Foulkes W.D., Smith I.E., Reis-Filho J.S. (2010). Triple-negative breast cancer. N. Engl. J. Med..

[19] Anders C.K., Carey L.A. (2009). Biology, metastatic patterns, and treatment of patients with triple-negative breast cancer. Clin. Breast Cancer.

[20] Colleoni M., Cole B.F., Viale G., Regan M.M., Price K.N., Maiorano E., Mastropasqua M.G., Crivellari D., Gelber R.D., Goldhirsch A. (2010). Classical cyclophosphamide, methotrexate, and fluorouracil chemotherapy is more effective in triple-negative, node-negative breast cancer: Results from two randomized trials of adjuvant chemoendocrine therapy for node-negative breast cancer. J. Clin. Oncol..

[21] Sorlie T., Perou C.M., Tibshirani R., Aas T., Geisler S., Johnsen H., Hastie T., Eisen M.B., van de Rijn M., Jeffrey S.S. (2001). Gene expression patterns of breast carcinomas distinguish tumor subclasses with clinical implications. Proc. Natl. Acad. Sci. USA.

[22] Garrido-Castro A.C., Lin N.U., Polyak K. (2019). Insights into Molecular Classifications of Triple-Negative Breast Cancer: Improving Patient Selection for Treatment. Cancer Discov..

[23] André F., Zielinski C.C. (2012). Optimal strategies for the treatment of metastatic triple-negative breast cancer with currently approved agents. Ann. Oncol..

[24] Sharma P. (2016). Biology and Management of Patients With Triple-Negative Breast Cancer. Oncologist.

[25] Pierobon M., Frankenfeld C.L. (2013). Obesity as a risk factor for triple-negative breast cancers: A systematic review and meta-analysis. Breast Cancer Res. Treat..

[26] Oualla K., Kassem L., Nouiakh L., Amaadour L., Benbrahim Z., Arifi S., Mellas N. (2020). Immunotherapeutic Approaches in Triple-Negative Breast Cancer: State of the Art and Future Perspectives. Int. J. Breast Cancer.

[27] Keenan T.E., Tolaney S.M. (2020). Role of Immunotherapy in Triple-Negative Breast Cancer. J. Natl. Compr. Cancer Netw..

[28] Topalian S.L., Hodi F.S., Brahmer J.R., Gettinger S.N., Smith D.C., McDermott D.F., Powderly J.D., Carvajal R.D., Sosman J.A., Atkins M.B. (2012). Safety, activity, and immune correlates of anti-PD-1 antibody in cancer. N. Engl. J. Med..

[29] Garon E.B., Rizvi N.A., Hui R., Leighl N., Balmanoukian A.S., Eder J.P., Patnaik A., Aggarwal C., Gubens M., Horn L. (2015). Pembrolizumab for the treatment of non-small-cell lung cancer. N. Engl. J. Med..

[30] Motzer R.J., Rini B.I., McDermott D.F., Redman B.G., Kuzel T.M., Harrison M.R., Vaishampayan U.N., Drabkin H.A., George S., Logan T.F. (2015). Nivolumab for Metastatic Renal Cell Carcinoma: Results of a Randomized Phase II Trial. J. Clin. Oncol..

[31] Rosenberg J.E., Hoffman-Censits J., Powles T., van der Heijden M.S., Balar A.V., Necchi A., Dawson N., O’Donnell P.H., Balmanoukian A., Loriot Y. (2016). Atezolizumab in patients with locally advanced and metastatic urothelial carcinoma who have progressed following treatment with platinum-based chemotherapy: A single-arm, multicentre, phase 2 trial. Lancet.

[32] Wolchok J.D., Chiarion-Sileni V., Gonzalez R., Rutkowski P., Grob J.J., Cowey C.L., Lao C.D., Wagstaff J., Schadendorf D., Ferrucci P.F. (2017). Overall Survival with Combined Nivolumab and Ipilimumab in Advanced Melanoma. N. Engl. J. Med..

[33] El-Khoueiry A.B., Sangro B., Yau T., Crocenzi T.S., Kudo M., Hsu C., Kim T.Y., Choo S.P., Trojan J., Welling T.H.R. (2017). Nivolumab in patients with advanced hepatocellular carcinoma (CheckMate 040): An open-label, non-comparative, phase 1/2 dose escalation and expansion trial. Lancet.

[34] Kim S.T., Cristescu R., Bass A.J., Kim K.M., Odegaard J.I., Kim K., Liu X.Q., Sher X., Jung H., Lee M. (2018). Comprehensive molecular characterization of clinical responses to PD-1 inhibition in metastatic gastric cancer. Nat. Med..

[35] Ribas A., Wolchok J.D. (2018). Cancer immunotherapy using checkpoint blockade. Science.

[36] Buchbinder E.I., Desai A. (2016). CTLA-4 and PD-1 pathways: Similarities, differences, and implications of their inhibition. Am. J. Clin. Oncol..

[37] Nasser N.J., Gorenberg M., Agbarya A. (2020). First line Immunotherapy for Non-Small Cell Lung Cancer. Pharmaceuticals.

[38] Wherry E.J., Kurachi M. (2015). Molecular and cellular insights into T cell exhaustion. Nat. Rev. Immunol..

[39] Paulsen E.E., Kilvaer T.K., Khanehkenari M.R., Al-Saad S., Hald S.M., Andersen S., Richardsen E., Ness N., Busund L.T., Bremnes R.M. (2017). Assessing PDL-1 and PD-1 in Non-Small Cell Lung Cancer: A Novel Immunoscore Approach. Clin. Lung Cancer.

[40] Turnis M.E., Andrews L.P., Vignali D.A. (2015). Inhibitory receptors as targets for cancer immunotherapy. Eur. J. Immunol..

[41] Fehrenbacher L., Spira A., Ballinger M., Kowanetz M., Vansteenkiste J., Mazieres J., Park K., Smith D., Artal-Cortes A., Lewanski C. (2016). Atezolizumab versus docetaxel for patients with previously treated non-small-cell lung cancer (POPLAR): A multicentre, open-label, phase 2 randomised controlled trial. Lancet.

[42] Denkert C., von Minckwitz G., Darb-Esfahani S., Lederer B., Heppner B.I., Weber K.E., Budczies J., Huober J., Klauschen F., Furlanetto J. (2018). Tumour-infiltrating lymphocytes and prognosis in different subtypes of breast cancer: A pooled analysis of 3771 patients treated with neoadjuvant therapy. Lancet. Oncol..

[43] Loi S., Drubay D., Adams S., Pruneri G., Francis P.A., Lacroix-Triki M., Joensuu H., Dieci M.V., Badve S., Demaria S. (2019). Tumor-Infiltrating Lymphocytes and Prognosis: A Pooled Individual Patient Analysis of Early-Stage Triple-Negative Breast Cancers. J. Clin. Oncol..

[44] Ibrahim E.M., Al-Foheidi M.E., Al-Mansour M.M., Kazkaz G.A. (2014). The prognostic value of tumor-infiltrating lymphocytes in triple-negative breast cancer: A meta-analysis. Breast Cancer Res. Treat..

[45] Liu S., Lachapelle J., Leung S., Gao D., Foulkes W.D., Nielsen T.O. (2012). CD8+ lymphocyte infiltration is an independent favorable prognostic indicator in basal-like breast cancer. Breast Cancer Res..

[46] Mittendorf E.A., Philips A.V., Meric-Bernstam F., Qiao N., Wu Y., Harrington S., Su X., Wang Y., Gonzalez-Angulo A.M., Akcakanat A. (2014). PD-L1 expression in triple-negative breast cancer. Cancer Immunol. Res..

[47] Miyashita M., Sasano H., Tamaki K., Hirakawa H., Takahashi Y., Nakagawa S., Watanabe G., Tada H., Suzuki A., Ohuchi N. (2015). Prognostic significance of tumor-infiltrating CD8+ and FOXP3+ lymphocytes in residual tumors and alterations in these parameters after neoadjuvant chemotherapy in triple-negative breast cancer: A retrospective multicenter study. Breast Cancer Res..

[48] Gatalica Z., Snyder C., Maney T., Ghazalpour A., Holterman D.A., Xiao N., Overberg P., Rose I., Basu G.D., Vranic S. (2014). Programmed cell death 1 (PD-1) and its ligand (PD-L1) in common cancers and their correlation with molecular cancer type. Cancer Epidemiol. Biomark. Prev..

[49] Luen S., Virassamy B., Savas P., Salgado R., Loi S. (2016). The genomic landscape of breast cancer and its interaction with host immunity. Breast.

[50] Hammond M.E., Hayes D.F., Dowsett M., Allred D.C., Hagerty K.L., Badve S., Fitzgibbons P.L., Francis G., Goldstein N.S., Hayes M. (2010). American Society of Clinical Oncology/College of American Pathologists guideline recommendations for immunohistochemical testing of estrogen and progesterone receptors in breast cancer (unabridged version). Arch. Pathol. Lab. Med..

[51] Goldhirsch A., Gelber R.D., Coates A.S. (2005). What are the long-term effects of chemotherapy and hormonal therapy for early breast cancer?. Nat. Clin. Pract. Oncol..

[52] Lehmann B.D., Bauer J.A., Chen X., Sanders M.E., Chakravarthy A.B., Shyr Y., Pietenpol J.A. (2011). Identification of human triple-negative breast cancer subtypes and preclinical models for selection of targeted therapies. J. Clin. Investig..

[53] Topalian S.L., Drake C.G., Pardoll D.M. (2015). Immune checkpoint blockade: A common denominator approach to cancer therapy. Cancer Cell.

[54] Herbst R.S., Soria J.-C., Kowanetz M., Fine G.D., Hamid O., Gordon M.S., Sosman J.A., McDermott D.F., Powderly J.D., Gettinger S.N. (2014). Predictive correlates of response to the anti-PD-L1 antibody MPDL3280A in cancer patients. Nature.

[55] Nanda R., Liu M.C., Yau C., Shatsky R., Pusztai L., Wallace A., Chien A.J., Forero-Torres A., Ellis E., Han H. (2020). Effect of Pembrolizumab Plus Neoadjuvant Chemotherapy on Pathologic Complete Response in Women With Early-Stage Breast Cancer: An Analysis of the Ongoing Phase 2 Adaptively Randomized I-SPY2 Trial. JAMA Oncol..

[56] Adams S., Loi S., Toppmeyer D., Cescon D.W., De Laurentiis M., Nanda R., Winer E.P., Mukai H., Tamura K., Armstrong A. (2019). Pembrolizumab monotherapy for previously untreated, PD-L1-positive, metastatic triple-negative breast cancer: Cohort B of the phase II KEYNOTE-086 study. Ann. Oncol..

[57] Winer E.P., Lipatov O., Im S.A., Goncalves A., Muñoz-Couselo E., Lee K.S., Schmid P., Tamura K., Testa L., Witzel I. (2021). Pembrolizumab versus investigator-choice chemotherapy for metastatic triple-negative breast cancer (KEYNOTE-119): A randomised, open-label, phase 3 trial. Lancet Oncol..

[58] Schmid P., Adams S., Rugo H.S., Schneeweiss A., Barrios C.H., Iwata H., Diéras V., Hegg R., Im S.A., Shaw Wright G. (2018). Atezolizumab and Nab-Paclitaxel in Advanced Triple-Negative Breast Cancer. N. Engl. J. Med..

[59] Emens L.A., Cruz C., Eder J.P., Braiteh F., Chung C., Tolaney S.M., Kuter I., Nanda R., Cassier P.A., Delord J.P. (2019). Long-term Clinical Outcomes and Biomarker Analyses of Atezolizumab Therapy for Patients With Metastatic Triple-Negative Breast Cancer: A Phase 1 Study. JAMA Oncol..

[60] Dirix L.Y., Takacs I., Jerusalem G., Nikolinakos P., Arkenau H.T., Forero-Torres A., Boccia R., Lippman M.E., Somer R., Smakal M. (2018). Avelumab, an anti-PD-L1 antibody, in patients with locally advanced or metastatic breast cancer: A phase 1b JAVELIN Solid Tumor study. Breast Cancer Res. Treat..

[61] Masuda H., Baggerly K.A., Wang Y., Zhang Y., Gonzalez-Angulo A.M., Meric-Bernstam F., Valero V., Lehmann B.D., Pietenpol J.A., Hortobagyi G.N. (2013). Differential response to neoadjuvant chemotherapy among 7 triple-negative breast cancer molecular subtypes. Clin. Cancer Res..

[62] Petrelli F., Coinu A., Borgonovo K., Cabiddu M., Ghilardi M., Lonati V., Barni S. (2014). The value of platinum agents as neoadjuvant chemotherapy in triple-negative breast cancers: A systematic review and meta-analysis. Breast Cancer Res. Treat..

[63] Wood D.E. (2019). National Comprehensive Cancer Network: NCCN. Clinical practice guidelines in oncology. Thorac. Surg. Clin..

[64] Huang M., O’Shaughnessy J., Zhao J., Haiderali A., Cortes J., Ramsey S., Briggs A., Karantza V., Aktan G., Qi C.Z. (2020). Evaluation of Pathologic Complete Response as a Surrogate for Long-Term Survival Outcomes in Triple-Negative Breast Cancer. J. Natl. Compr. Cancer Netw..

[65] von Minckwitz G., Schneeweiss A., Loibl S., Salat C., Denkert C., Rezai M., Blohmer J.U., Jackisch C., Paepke S., Gerber B. (2014). Neoadjuvant carboplatin in patients with triple-negative and HER2-positive early breast cancer (GeparSixto; GBG 66): A randomised phase 2 trial. Lancet Oncol..

[66] Emens L.A., Middleton G. (2015). The interplay of immunotherapy and chemotherapy: Harnessing potential synergies. Cancer Immunol. Res..

[67] Kulangara K., Zhang N., Corigliano E., Guerrero L., Waldroup S., Jaiswal D., Ms M.J., Shah S., Hanks D., Wang J. (2019). Clinical Utility of the Combined Positive Score for Programmed Death Ligand-1 Expression and the Approval of Pembrolizumab for Treatment of Gastric Cancer. Arch. Pathol. Lab. Med..

[68] Franzoi M.A., de Azambuja E. (2020). Atezolizumab in metastatic triple-negative breast cancer: IMpassion130 and 131 trials—How to explain different results?. ESMO Open.

[69] Schmid P., Cortes J., Pusztai L., McArthur H., Kümmel S., Bergh J., Denkert C., Park Y.H., Hui R., Harbeck N. (2020). Pembrolizumab for Early Triple-Negative Breast Cancer. N. Engl. J. Med..

[70] Suppan C., Balic M. (2020). Treatment options in early triple-negative breast cancer. Memo.

[71] Cortes J., Cescon D.W., Rugo H.S., Nowecki Z., Im S.A., Yusof M.M., Gallardo C., Lipatov O., Barrios C.H., Holgado E. (2020). Pembrolizumab plus chemotherapy versus placebo plus chemotherapy for previously untreated locally recurrent inoperable or metastatic triple-negative breast cancer (KEYNOTE-355): A randomised, placebo-controlled, double-blind, phase 3 clinical trial. Lancet.

[72] McArthur H.L., Ignatiadis M., Guillaume S., Bailey A., Martinez J.L., Brandao M., Metzger O., Lai C., Fumagalli D., Daly F. (2019). ALEXANDRA/IMpassion030: A phase III study of standard adjuvant chemotherapy with or without atezolizumab in early-stage triple-negative breast cancer. J. Clin. Oncol..

[73] Mittendorf E.A., Zhang H., Barrios C.H., Saji S., Jung K.H., Hegg R., Koehler A., Sohn J., Iwata H., Telli M.L. (2020). Neoadjuvant atezolizumab in combination with sequential nab-paclitaxel and anthracycline-based chemotherapy versus placebo and chemotherapy in patients with early-stage triple-negative breast cancer (IMpassion031): A randomised, double-blind, phase 3 trial. Lancet.

[74] Geyer C.E., Loibl S., Rastogi P., Seiler S., Costantino J.P., Vijayvergia N., Cortazar P., Lucas P.C., Denkert C., Mamounas E.P. (2018). NSABP B-59/GBG 96-GeparDouze: A randomized double-blind phase III clinical trial of neoadjuvant chemotherapy (NAC) with atezolizumab or placebo in Patients (pts) with triple negative breast cancer (TNBC) followed by adjuvant atezolizumab or placebo. J. Clin. Oncol..

[75] Vikas P., Borcherding N., Zhang W. (2018). The clinical promise of immunotherapy in triple-negative breast cancer. Cancer Manag. Res..

[76] Selby M.J., Engelhardt J.J., Quigley M., Henning K.A., Chen T., Srinivasan M., Korman A.J. (2013). Anti-CTLA-4 antibodies of IgG2a isotype enhance antitumor activity through reduction of intratumoral regulatory T cells. Cancer Immunol. Res..

[77] Larkin J., Hodi F.S., Wolchok J.D. (2015). Combined Nivolumab and Ipilimumab or Monotherapy in Untreated Melanoma. N. Engl. J. Med..

[78] Hellmann M.D., Ciuleanu T.E., Pluzanski A., Lee J.S., Otterson G.A., Audigier-Valette C., Minenza E., Linardou H., Burgers S., Salman P. (2018). Nivolumab plus Ipilimumab in Lung Cancer with a High Tumor Mutational Burden. N. Engl. J. Med..

[79] Li Z., Qiu Y., Lu W., Jiang Y., Wang J. (2018). Immunotherapeutic interventions of Triple Negative Breast Cancer. J. Transl. Med..

[80] Thomas R., Al-Khadairi G., Decock J. (2020). Immune Checkpoint Inhibitors in Triple Negative Breast Cancer Treatment: Promising Future Prospects. Front. Oncol..

[81] Jiao S., Xia W., Yamaguchi H., Wei Y., Chen M.K., Hsu J.M., Hsu J.L., Yu W.H., Du Y., Lee H.H. (2017). PARP Inhibitor Upregulates PD-L1 Expression and Enhances Cancer-Associated Immunosuppression. Clin. Cancer Res..

[82] Mina L.A., Lim S., Bahadur S.W., Firoz A.T. (2019). Immunotherapy for the Treatment of Breast Cancer: Emerging New Data. Breast Cancer.

[83] Brahmer J.R., Lacchetti C., Schneider B.J., Atkins M.B., Brassil K.J., Caterino J.M., Chau I., Ernstoff M.S., Gardner J.M., Ginex P. (2018). Management of Immune-Related Adverse Events in Patients Treated With Immune Checkpoint Inhibitor Therapy: American Society of Clinical Oncology Clinical Practice Guideline. J. Clin. Oncol..

[84] Ahmed F.S., Gaule P., McGuire J., Patel K., Blenman K., Pusztai L., Rimm D.L. (2020). PD-L1 Protein Expression on Both Tumor Cells and Macrophages are Associated with Response to Neoadjuvant Durvalumab with Chemotherapy in Triple-negative Breast Cancer. Clin. Cancer Res..

[85] Savas P., Virassamy B., Ye C., Salim A., Mintoff C.P., Caramia F., Salgado R., Byrne D.J., Teo Z.L., Dushyanthen S. (2018). Single-cell profiling of breast cancer T cells reveals a tissue-resident memory subset associated with improved prognosis. Nat. Med..

[86] Thomas A., Routh E.D., Pullikuth A., Jin G., Su J., Chou J.W., Hoadley K.A., Print C., Knowlton N., Black M.A. (2018). Tumor mutational burden is a determinant of immune-mediated survival in breast cancer. Oncoimmunology.

[87] Zacharakis N., Chinnasamy H., Black M., Xu H., Lu Y.C., Zheng Z., Pasetto A., Langhan M., Shelton T., Prickett T. (2018). Immune recognition of somatic mutations leading to complete durable regression in metastatic breast cancer. Nat. Med..

[88] Tran E., Turcotte S., Gros A., Robbins P.F., Lu Y.C., Dudley M.E., Wunderlich J.R., Somerville R.P., Hogan K., Hinrichs C.S. (2014). Cancer immunotherapy based on mutation-specific CD4+ T cells in a patient with epithelial cancer. Science.

[89] Cortes-Ciriano I., Lee S., Park W.Y., Kim T.M., Park P.J. (2017). A molecular portrait of microsatellite instability across multiple cancers. Nat. Commun..

[90] Nolan E., Savas P., Policheni A.N., Darcy P.K., Vaillant F., Mintoff C.P., Dushyanthen S., Mansour M., Pang J.B., Fox S.B. (2017). Combined immune checkpoint blockade as a therapeutic strategy for BRCA1-mutated breast cancer. Sci. Transl. Med..

[91] Palaia I., Tomao F., Sassu C.M., Musacchio L., Benedetti Panici P. (2020). Immunotherapy For Ovarian Cancer: Recent Advances And Combination Therapeutic Approaches. OncoTargets Ther..

[92] Ramos-Casals M., Brahmer J.R., Callahan M.K., Flores-Chávez A., Keegan N., Khamashta M.A., Lambotte O., Mariette X., Prat A., Suárez-Almazor M.E. (2020). Immune-related adverse events of checkpoint inhibitors. Nat. Rev. Dis. Primers.

[93] Yoest J.M. (2017). Clinical features, predictive correlates, and pathophysiology of immune-related adverse events in immune checkpoint inhibitor treatments in cancer: A short review. Immunotargets Ther..

[94] Kanjanapan Y., Day D., Butler M.O., Wang L., Joshua A.M., Hogg D., Leighl N.B., Razak A.R.A., Hansen A.R., Boujos S. (2019). Delayed immune-related adverse events in assessment for dose-limiting toxicity in early phase immunotherapy trials. Eur. J. Cancer.

[95] Kartolo A., Sattar J., Sahai V., Baetz T., Lakoff J.M. (2018). Predictors of immunotherapy-induced immune-related adverse events. Curr. Oncol..

[96] Eun Y., Kim I.Y., Sun J.M., Lee J., Cha H.S., Koh E.M., Kim H., Lee J. (2019). Risk factors for immune-related adverse events associated with anti-PD-1 pembrolizumab. Sci. Rep..

[97] Weber J.S., Hodi F.S., Wolchok J.D., Topalian S.L., Schadendorf D., Larkin J., Sznol M., Long G.V., Li H., Waxman I.M. (2017). Safety Profile of Nivolumab Monotherapy: A Pooled Analysis of Patients With Advanced Melanoma. J. Clin. Oncol..

[98] Horvat T.Z., Adel N.G., Dang T.O., Momtaz P., Postow M.A., Callahan M.K., Carvajal R.D., Dickson M.A., D’Angelo S.P., Woo K.M. (2015). Immune-Related Adverse Events, Need for Systemic Immunosuppression, and Effects on Survival and Time to Treatment Failure in Patients With Melanoma Treated With Ipilimumab at Memorial Sloan Kettering Cancer Center. J. Clin. Oncol..

[99] Arbour K.C., Mezquita L., Long N., Rizvi H., Auclin E., Ni A., Martínez-Bernal G., Ferrara R., Lai W.V., Hendriks L.E.L. (2018). Impact of Baseline Steroids on Efficacy of Programmed Cell Death-1 and Programmed Death-Ligand 1 Blockade in Patients With Non-Small-Cell Lung Cancer. J. Clin. Oncol..

[100] Maher V.E., Fernandes L.L., Weinstock C., Tang S., Agarwal S., Brave M., Ning Y.M., Singh H., Suzman D., Xu J. (2019). Analysis of the Association Between Adverse Events and Outcome in Patients Receiving a Programmed Death Protein 1 or Programmed Death Ligand 1 Antibody. J. Clin. Oncol..

[101] Marra A., Viale G., Curigliano G. (2019). Recent advances in triple negative breast cancer: The immunotherapy era. BMC Med..

